# Dataset of socio-economic and waste collection indicators for Portugal at municipal level

**DOI:** 10.1016/j.dib.2018.12.069

**Published:** 2018-12-28

**Authors:** Verónica Oliveira, Vitor Sousa, Celia Dias-Ferreira

**Affiliations:** a Research Centre for Natural Resources, Environment and Society (CERNAS), College of Agriculture (ESAC), Polytechnic Institute of Coimbra, Bencanta, 3045-601 Coimbra, Portugal; b Materials and Ceramic Engineering Department, CICECO, University of Aveiro, Campus Universitário de Santiago, 3810-193 Aveiro, Portugal; c CERIS, Department of Civil Engineering, Architecture and GeoResources, Tecnico Lisboa - IST, Av. Rovisco Pais, Lisbon, Portugal

## Abstract

This data article presents demographic, socio-economic and waste-related data at municipal level for Portugal. The dataset includes raw data collected from 4 main sources: (i) the annual reports of waste management companies; (ii) the database of the Portuguese water, sanitation and waste regulatory entity; (iii) the Portuguese Environmental Agency; and (iv) national statistical data. Relevant indicators for waste generation and for the separate collection of waste are proposed and calculated using the raw data. The dataset comprises municipalities with high, medium and low separate collection yields, providing socio-economic and waste infrastructures data that can be used for benchmarking. The dataset can also be used to define a baseline against which the progress of the collection of packaging waste can be assessed over time, or else serve as input to mathematical models predicting waste generation and collection. Moreover, data can serve as the base to calculate new waste-related indicators. In addition to being a valuable input to the waste topic, the dataset can also be used in a large range of other topics where demographic and socio-economic parameters are relevant. The data presented herein are associated with the research articles “Model for the separate collection of packaging waste in Portuguese low-performing recycling regions” [1] and “Artificial neural network modelling of the amount of separately-collected household packaging waste” [2].

**Specifications table**

**Table:** 

Subject area	*Environmental science*
More specific subject area	*Waste Management and Disposal*
Type of data	*Table*
How data were acquired	*Data were acquired from the national statistical databases, from reports of waste management companies, from regulatory entities and Portuguese environmental agency.*
Data format	*Raw, Calculated*
Experimental factors	*The raw data collected were organized in spreadsheets; the information was organised by alphabetic order, based on the name of the municipality. For the indicator “proportion of population of a certain age” the information was grouped into classes, which aggregated three or more smaller classes.*
Experimental features	*Calculation of waste indicators and socio-economic indicators*
Data source location	*Portugal*
Data accessibility	*Data are provided in the article*
*Related research article*	i) *V. Oliveira, V. Sousa, J. M. Vaz, C. Dias-Ferreira, Model for the separate collection of packaging waste in Portuguese low-performing recycling regions, J. Environ. Manage, 216 (2018) 13–24* ii) *V. Oliveira, V. Sousa, C. Dias-Ferreira, Artificial neural network modelling of the amount of separately-collected household packaging waste, J. Cleaner Production, 210 (2019) 401–409.*

**Value of the data**

• The dataset comprises municipalities with high, medium and low separate collection yields, providing data on demographic and socio-economic parameters and waste infrastructures that can be used for benchmarking.
• Dataset can be used as a baseline against which the progress of the collection of packaging waste can be assessed in time.
• This dataset can be used to calculate new indicators on the separate collection of waste.
• The dataset can be used as input to models predicting waste generation and collection.
• The dataset is valuable in the assessment of situations and case studies influenced by socio economic parameters.

## 1 Data

The demographic and socio-economic indicators and corresponding raw data for 243 municipalities in Portugal are included as Supplementary material (Tables 1-a–6).

For each municipality, the surface area (km^2^), the total population (inhab.), the proportion of population living in urbanized areas of different size (<2000 inhab.; between 2000–4999 inhab.; 5000–9999 inhab.; >10,000 inhab.), the proportion of population of a certain age (<20 years old; 20–40 years old; 40–65 years old; >65 years old) and the degree of urbanisation are listed in Supplementary Table 1-a. The percentage of the population over 15-year-old with a certain level of education (without education or with the 1^st^ cycle; with the 2^nd^ cycle, with 3^rd^ cycle, at secondary level, with high school) and the average number of school years attended by the population are presented in Supplementary Table 1-b.

The proportion of population living in urbanized areas of different size (in Table 1-a) was based on the raw data presented in Supplementary Table 1-c. The proportion of population of a certain age (in Table 1-a) was computed from the raw data compiled in Supplementary Table 1-d. The degree of urbanisation (in Table 1-a) was based on the urbanisation level of the parishes and corresponding population, which is shown in Supplementary Table 1-e.

The Supplementary Table 2 lists data related to economic deprivation, namely the criminality, unemployment and incidence of mandatory notification diseases ratios (raw data), as well as the calculated value for the deprivation index. Supplementary Table 3 shows the average values for each municipality of the family income per year (€), the purchase power per capita, and the purchase power index (%).

Indicators and raw data related to the amount of waste are listed in Supplementary Table 4. It comprises the following: unsorted waste collected (total; per inhabitant per year), separately collected packaging waste (total; per inhabitant per year) and separate collection of packaging waste rate (%).

Indicators related to the waste collection infrastructures are presented in Supplementary Table 5-a and b. The Supplementary Table 5-a includes the containers available for unsorted waste collection (total; per inhabitant; per area) and the population covered per unsorted waste container. The Supplementary Table 5-b comprises the containers available for collection of packaging waste (total; per inhabitant; per area), the population served per bring-bank, civic amenity drop-off sites (total and per surface area), and the entities responsible for the waste management in each municipality. In Supplementary Table 6 are listed the indicators related to the waste collection service, namely relative accessibility to bring-banks and accessibility to separate collection services (%).

## 2 Experimental design, materials and methods

The data presented in this data article were acquired from 4 main sources: (i) the annual reports of waste management companies [3], [4]; (ii) the Portuguese water, sanitation and waste regulatory entity databases [5]; (iii) the Portuguese environmental agency [6]; and (iv) national statistics [7], [8], [9], [10], [11], [12], [13], [14], [15], [16], [17].

Based on the data, relevant indicators were selected following the recommendations in Oliveira et al. [1]. The raw data collected were organized in spreadsheets (MS Excel) and the calculation of indicators were carried out using the embedded tools in the software. The level of data disaggregation was the “municipality” for all data, except for the “degree of urbanisation”, for which data at the parish-level was used. The data for all the parishes in a given municipality were then integrated to construct the indicator “degree of urbanisation” for a specific municipality. The methodology of calculation of the indicators followed the methodology described in Oliveira et al. [1].

Data refers always to annual values. In some situations, the sources for the same type of data were different because of the required disaggregation level. For instance, Pordata [7] was used to retrieve the population in each municipality for the year 2015. However, for calculating the degree of urbanisation the population at the parish could not be obtained from the same data source, because it referred only to the municipality level. In this situation, the most recent data available for the parish was retrieved from Statistics Portugal [12] and refers to the year 2011. In each case, the sources used are clearly identified in the tables.
